# Metadevices with Potential Practical Applications

**DOI:** 10.3390/molecules24142651

**Published:** 2019-07-22

**Authors:** Yafei Li, Jiangtao Lv, Qiongchan Gu, Sheng Hu, Zhigang Li, Xiaoxiao Jiang, Yu Ying, Guangyuan Si

**Affiliations:** 1 College of Information Science and Engineering, Northeastern University, Shenyang 110004, China; 2 College of Information & Control Engineering, Shenyang Jianzhu University, Shenyang 110168, China; 3 Melbourne Centre for Nanofabrication, Clayton, Victoria 3168, Australia

**Keywords:** metamaterials, near-field energy transfer, mirrors, nanobiosensors, detectors

## Abstract

Metamaterials are “new materials” with different superior physical properties, which have generated great interest and become popular in scientific research. Various designs and functional devices using metamaterials have formed a new academic world. The application concept of metamaterial is based on designing diverse physical structures that can break through the limitations of traditional optical materials and composites to achieve extraordinary material functions. Therefore, metadevices have been widely studied by the academic community recently. Using the properties of metamaterials, many functional metadevices have been well investigated and further optimized. In this article, different metamaterial structures with varying functions are reviewed, and their working mechanisms and applications are summarized, which are near-field energy transfer devices, metamaterial mirrors, metamaterial biosensors, and quantum-cascade detectors. The development of metamaterials indicates that new materials will become an important breakthrough point and building blocks for new research domains, and therefore they will trigger more practical and wide applications in the future.

## 1. Introduction

Metamaterial is a new academic term in the field of physics in the 21st century. It has become a nova quickly due to its unique physical properties that natural materials do not possess. The characteristics of metamaterials include ultra-high equivalent refractive indices [1,2], negative electromagnetic parameters [3] and negative refractive indices [4]. Based on these special physical properties, there are various metamaterial devices which have been well investigated and developed, including photonic crystals [5], left-handed materials [6], invisibility cloaking [7] and absorbent devices [8]. Here, we review different types of important metadevices, including their working mechanisms, which show abundant application directions of metamaterials.

According to Planck′s law [9], blackbody radiation generates a large amount of thermal energy in the far-field, and then some far-field energy transfer devices have come into being [10]. When the characteristic size of the object is comparable to the wavelength of thermal radiation, the Planck blackbody radiation theory is not valid anymore [11]. Section 2 introduces near-field energy transfer devices that mainly include a microsphere and a flat substrate, which can use evanescent waves to transfer broadband thermal energy and significantly improve the transmission efficiency of the near-field energy. By measuring the conductance between the microsphere and the plate, it is found that less energy is transferred with increasing distance between them. Research on near-field energy transfer has critical guiding significance for the application of many other technologies in the future, such as conversion of thermal radiation [12], cooling radiation [13], thermal infrared imaging [14] and thermal assisted magnetic recording [15].

Mirrors are commonly used in daily life, and they can date back to more than 8000 years ago [16]. Although mirrors are common, the peculiar properties and functions of metamaterial mirrors deserve researchers’ attention. They play a key role in optics, but when the semiconductor layer comes into contact with the metal layer, the incident light is reflected, and the electric field in the surrounding area is lowered. When a beam is reflected by a mirror, the phase is reversed, and the standing wave near the mirror is weakened, which affects the overall thickness of the optical devices. In Section 3, we review special metamaterial mirrors (MMs) that can enhance light interaction with thin films and act as effective magnetic mirrors. Such MMs can be expected to provide a basis for solar cells that respond to randomly polarized light to improve light absorption and photocurrent, dramatically increasing the device efficiency. The design of MMs draws on the concept of metasurfaces. Compared with traditional optical components, metasurfaces are two-dimensional subwavelength structures, and they show different characteristics to the incident light [17,18,19,20,21,22,23,24,25,26,27,28,29,30,31,32,33]. Various devices have been realized by using metasurfaces, such as Hilbert space-filling curves [24], mushroom-like nanodisks [25], planar chiral structures [26], fish scale structures [28,29], and complex nanoantenna arrays [30,31,32]. 

Biosensors based on surface plasmon resonances (SPRs) [34,35,36,37] have become an important tool to detect the interaction between biomolecules [38]. Although they have irreplaceable advantages in providing data information, molecular affinity, and changing the speed of molecular motion, some certain information of unknown molecules is still not able to be sensed [35]. Section 4 presents label-free metamaterial biosensors based on surface-enhanced Raman scattering (SERS) that can detect conformational vibration and small molecules, which select G-quadruplexes (G4) moieties as plasmonic response regulators and captors for sensing of malachite green (MG) [39,40,41,42]. Metamaterials are complementary to new sensing technologies and conventional plasmon sensing techniques [43,44,45,46,47]. By changing the geometry of metamaterials, the electromagnetic response can be adjusted from the terahertz (THz) regime [48,49] to visible-near-infrared region [50,51]. Metamaterials combined with biomolecules show powerful functions, which can not only accurately express the structure state of biomolecules, but also possess great application potential in the discovery of new drugs and detection of cancer biomarkers with ultra-high sensitivities. Metamaterials have also been used to recognize fingerprints and detect organic molecules, DNA [43], and proteins [37,44,52] in response to specific chemicals or biological molecular vibration patterns.

Section 5 mainly focuses on the characterization of split-ring resonant metamaterial detectors with different periods [53] and polarization-dependent properties [54] based on quantum-cascade structures. The metamaterial structure was fabricated on the top layer of metal contact, which was used to couple normal incidence radiation resonantly to the inter-sub-band transitions and form a double-metal waveguide together with the metallic ground plane. This structure can significantly enhance the photocurrent response obtained at the metamaterial resonances. The high-performance semiconductor inter-sub-band infrared photodetectors are highly needed for a variety of applications, due to their capabilities of flexible energy-band tailoring and ultrahigh operation speeds [55,56,57]. Quantum cascade lasers (QCLs) provide dozens of milliwatts power over a wide wavelength range [58,59], and they can be combined with standard detectors to probe chemical detection [60,61]. Based on the development of QCLs, the new type of quantum cascade detectors (QCDs) with excellent performance have been proposed and studied in recent years [62], such as low noise photovoltaic operation mode QCDs [63], wide covering wavelengths QCDs [64,65,66], and high operating temperature QCDs [67,68,69].

## 2. Near-Field Energy Transfer Devices

**Figure 1:**
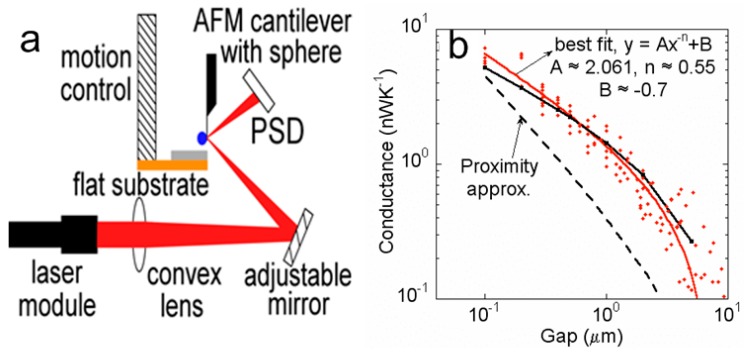
(**a**) Schematic of experimental setup for near-field energy transfer study. (**b**) Near-field conductance as a function of gap. Reproduced with permission from [70]. Copyright American Physical Society, 2008.

Narayanaswamy and coworkers have studied near-field energy transfer between a microsphere and a flat substrate using the bi-material atomic force microscope (AFM) cantilever as a thermal sensor [70]. The experimental device is shown in Figure 1a. A silica sphere with the diameter of ~50 µm is attached to the overhanging tip of the cantilever which can keep the cantilever perpendicular to the substrate for decreasing the influence of dispersion and electrostatic forces. The position sensitive detector (PSD) and sum signals represent the position of the spot formed by the reflected light. In the experiment, different distances of the microsphere-substrate and near-field conductance curves are measured in Figure 1b. The red diamond mark points are the measured data point, and the maximum conductance is 6 nW/k. In addition, it is found that the conductance between the two spheres satisfies Ax^−n^, where A is the coefficient, x is the distance between the two spheres, and n is an exponent less than 1 [71,72]. For the microsphere-substrate model, the conductance should meet the relation of Ax^−n^ + B, where B < 0. Fitted values of n, A, and B are 0.55, 2.061, and −0.7, respectively. The data points are distributed on both sides of the fitting curve. The black dotted line is the approximate prediction, which is below the measured values, showing the approximation method is not suitable for near-field radiation when the diameter of the microsphere or the gap is large [71]. The conductance of two microspheres multiplied by two is the black line with black squares curve. The measurement of the radiation transfer between the microsphere-substrate shows that there is a strong near field effect and the heat transfer is enhanced.

**Figure 2:**
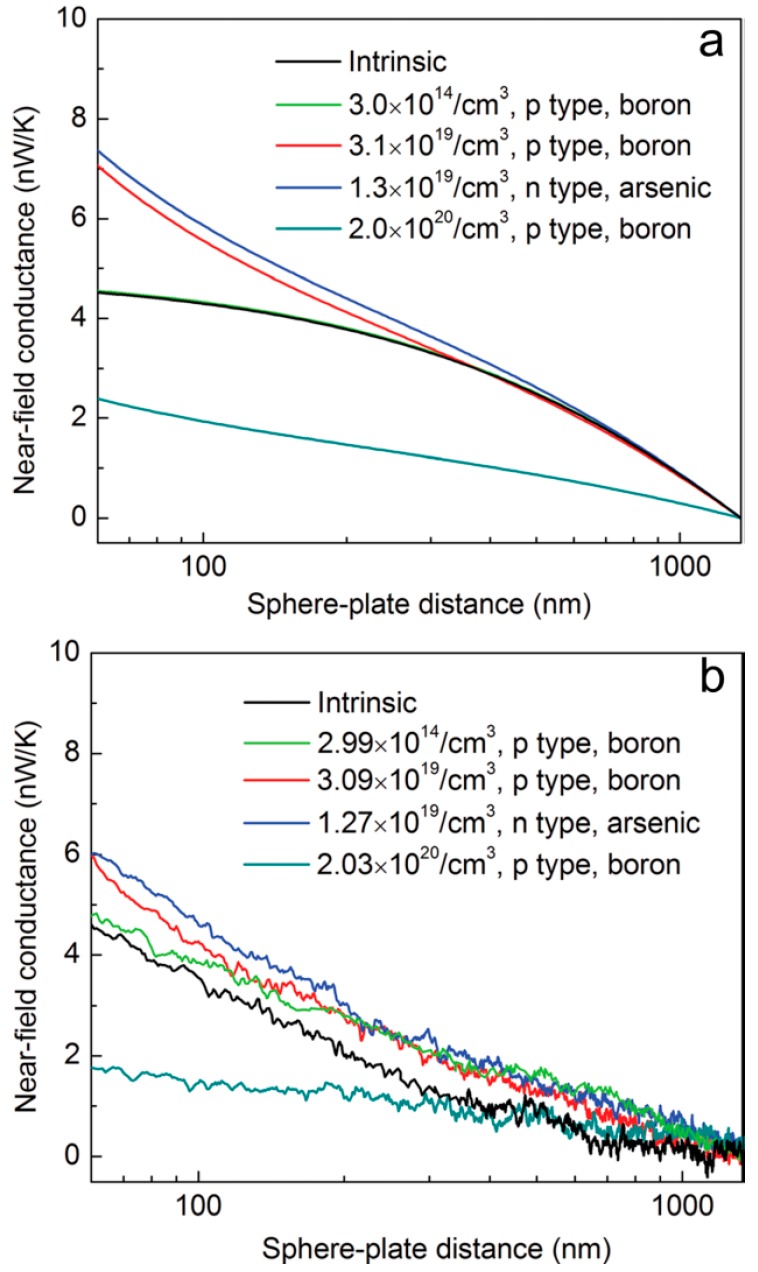
Near field conductance curves corresponding to five silicon samples with different carrier concentrations. (**a**) Theoretical calculation and (**b**) experimental measurement. Reproduced with permission from [73]. Copyright AIP Publishing, 2013.

Subsequently, the structure is optimized by a doped silicon substrate to change the carrier concentration and regulate the near field radiation [73]. In thermal equilibrium, electrons or ions that move freely in a material form electric current and radiate electromagnetic waves [74]. Although the doped silicon plate does not produce SPRs, it generates evanescent waves that can enhance the near-field radiation [75]. The experimental structure is similar to Figure 1a. A nanosphere with ~100 µm diameter is attached to the tip of the bi-material rectangle cantilever. Silicon samples with different carrier concentrations are placed on the flat substrate. The near-field conductance between silicon samples with five different carrier concentrations and nanospheres are plotted in Figure 2. In Figure 2a, the black curve shows the conductance of the intrinsic silicon sample. The carrier concentration of p-type doped silicon are 3.0 × 10^14^/cm^3^ (green curve), 3.0 × 10^19^/cm^3^ (red curve), 2.0 × 10^20^/cm^3^ (cyan curve), respectively. In addition, the near-field conductance decreases sharply as the distance increases when carrier concentration is 2.0 × 10^20^/cm^3^. This is because the peak of the local density of the states (LDOS) [76] of doped silicon deviates from the LDOS peak of the nanosphere when carrier concentration is too high [73]. For the n-type sample with a carrier concentration of 1.3 × 10^19^/cm^3^ (blue curve), its conductance exceeds that of the intrinsic silicon. In Figure 2b, the measured near-field conductance curves of the five samples are generally consistent with the theoretical calculations (Figure 2a). As the distance between the microsphere and the plane increases, the conductance of the five samples decreases. However, the conductance measured in the experiment is slightly less than the theoretical calculation due to signal noise and cantilever calibration error. P-type samples with carrier concentration of 2.99 × 10^14^/cm^3^ (green curve) and 3.09 × 10^19^/cm^3^ (red curve) show higher near field conductance than the intrinsic silicon. Therefore, when the distance between the microsphere and the plane is 60 nm, the near-field conductance changes from 2 nW/K to 6 nW/K by varying the carriers concentration in silicon. The tunable near field conductance provides a reference for the development of thermal photoelectric devices and thermal management systems [73].

**Figure 3:**
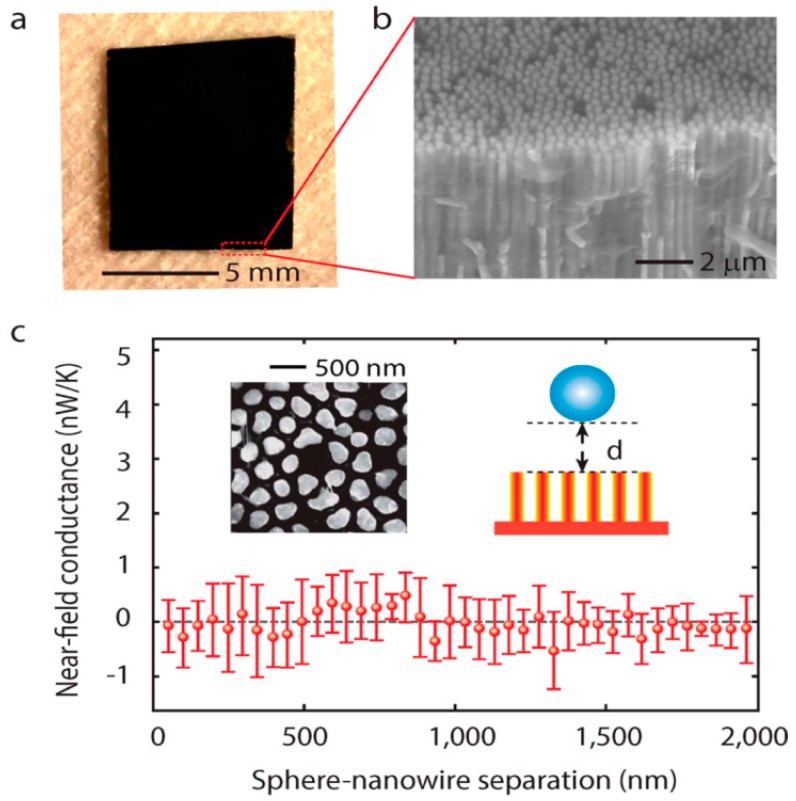
Optical and SEM of nickel nanowires arrays and proof of their loss-free property. (**a**) Optical and (**b**) SEM image of the nickel nanowires array. (**c**) The near field conductance as a function of distance between the nanosphere and nanowires. The top left inset is top view SEM of nanowires array. Reproduced with permission from [84]. Copyright American Chemical Society, 2015.

**Figure 4:**
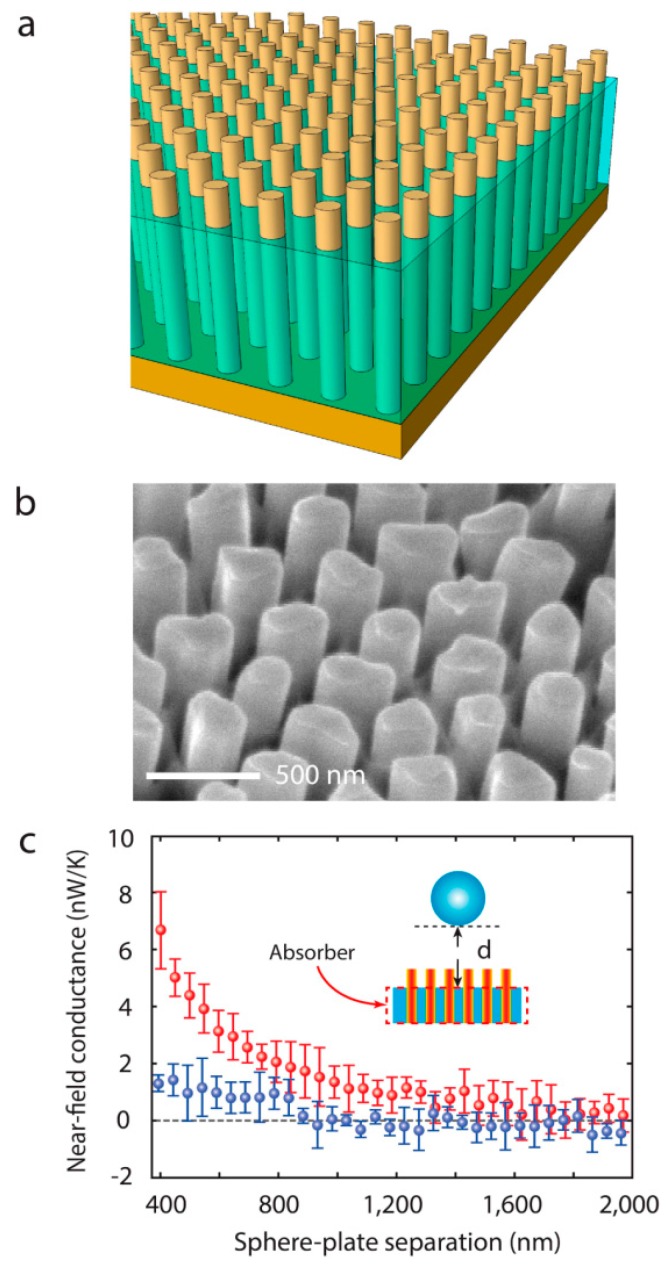
Demonstration of near-field energy transfer. (**a**) Schematic and (**b**) SEM image of the exposed nickel nanowires array. (**c**) The near field thermal conductance as a function of the distance between the nanosphere and AAO template with (red dots) and without (blue dots) exposed nanowires. Reproduced with permission from [84]. Copyright American Chemical Society, 2015.

As an important branch of electromagnetic metamaterials, hyperbolic metamaterials (HMs) have become the focus of research due to their unique near-field electromagnetic manipulation characteristics [77]. By changing the size and geometry of HMs, the intensity and direction of the surface plasmon polaritons (SPPs) can be precisely controlled [78,79]. Metamaterials composed of metal wire arrays (MWAs) can produce hyperbolic dispersion in a broadband range and transmit energy without loss [80]. Vertical nickel nanowires can be fabricated on anodic aluminum oxide (AAO) nanopore templates [81,82,83,84] and Figure 3a,b shows the optical and scanning electron microscopy (SEM) images of the sample, respectively. In this experiment, the near-field radiation between a SiO_2_ nanosphere and nanowires array on a 30 nm thick nickel film is measured. The nanowires have 10 µm length and 300 nm diameter. The distance between the nanowires is ~100 nm (top left inset of Figure 3c). Figure 3c plots the conductance between the nanosphere and nanowires, which shows that there is almost no heat transfer between them. The heat loss of nanowires is so low in the heat transfer process that it can be ignored. In addition, the incident light propagates to the nickel plate and then is reflected. Even if the distance between them is less than 50 nm, there is almost no energy transfer. Therefore, the nickel nanowires can be used as a lossless thermal radiation waveguide grown on a loss plane. The exposed portion of the nickel nanowire array is approximately 400 nm as shown in Figure 4a,b, and the rest enclosed by a red dotted box is the AAO template as shown in Figure 4c inset. The distance between the nanosphere and AAO plane is about 400 nm and we can see the near field energy transfer effect is increased. When the distance is very small, which is close to the exposed part of nanowires (~400 nm), measured thermal conductance is 7 nW/K (red dot). 

## 3. Metamaterial Mirrors 

**Figure 5:**
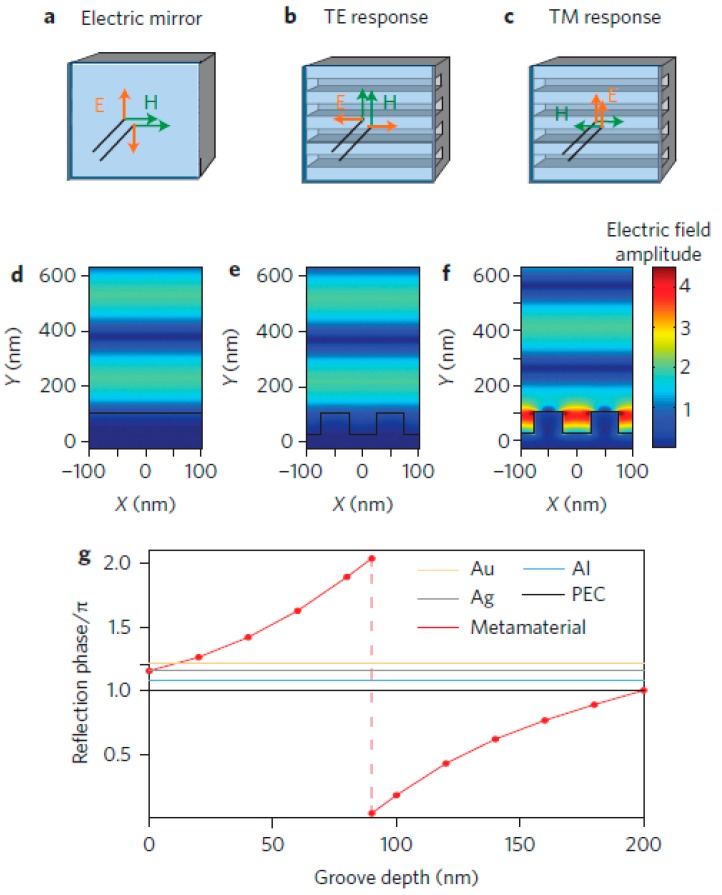
(**a**) Metamaterial mirrors (MMs) as electric mirror. (**b**) Flip the electric field of transverse electric (TE)-polarized light on reflection and as magnetic mirror. (**c**) Flip the magnetic field of transverse magnetic (TM)-polarized light. (**d**–**f**) The electric field distributions of the incident light when it is reflected from (**d**) a conventional planar silver mirror and (**e**) grooved silver MMs with TE polarized light and (**f**) TM polarized light. (**g**) The effect of groove depth on the reflection phase of different materials. Reproduced with permission from [86]. Copyright Springer Nature, 2014.

The generation of standing waves makes the electric field intensity on the surface decrease. This phenomenon is undesirable in structures that use metal films as a photoelectric device since it limits the distance between the metal and the semiconductor layer and affects the overall thickness of the device. When the incident light hits a mirror, the reflected light will also generate an electric field that responds to the incident, weakening the surrounding electric field (Figure 5a). The appearance of the magnetic mirror [85] can solve the above problem perfectly, which has both variable reflection phase and high surface electric field. The tunability of reflection phase can also be used to adjust the standing waves in planar devices to achieve better interaction between light and metals. It can reverse the magnetic field of the incident light rather than reflect electric energy (Figure 5a–c). The surface electric field of highly conductive metal is zero, which makes the surface impedance very small and inverts the reflected phase φ. Based on this principle, the nanoscale slot array forms metamaterial mirrors (MMs), and the performance is measured by the surface impedance. The grooves cause current on the metal surface to zigzag, changing the surface impedance and reflected phase. Compared with a smooth metal surface, changing the width of grooves can make the surface impedance various. When the impedance is large, reflected waves are generated in the mirror to represent the characteristics of the electromagnetic mirror.

Figure 5d shows that the conventional planar silver mirror reflects incident light of 600 nm and generates standing waves and a small surface electric field. The phenomenon in Figure 5e is similar to Figure 5d that the groove array with a width of 50 nm and a depth of 80 nm is irradiated by transverse electric (TE) polarized light. When irradiated with transverse magnetic (TM) polarized light, the phenomenon in Figure 5f reveals. Figure 5g plots the reflection phases of several materials (Au, Al, Ag, perfect electrical conductor (PEC)) with different groove depths, compared with metamaterials. The reflection phase is slightly larger than π. However, the reflection phase does not change with increasing groove depths. The reflection phase of the MMs varies approximately linearly because it is controlled by the gap SPPs that can select phase as it travels along the groove. As the depth of groove varies from 80 nm to 200 nm, the reflection phase of MMs changes from 0 to π, which makes the mirror change from perfect electric mirror to perfect magnetic mirror. Therefore, the groove depth affects the performance of the magnetic mirror.

**Figure 6:**
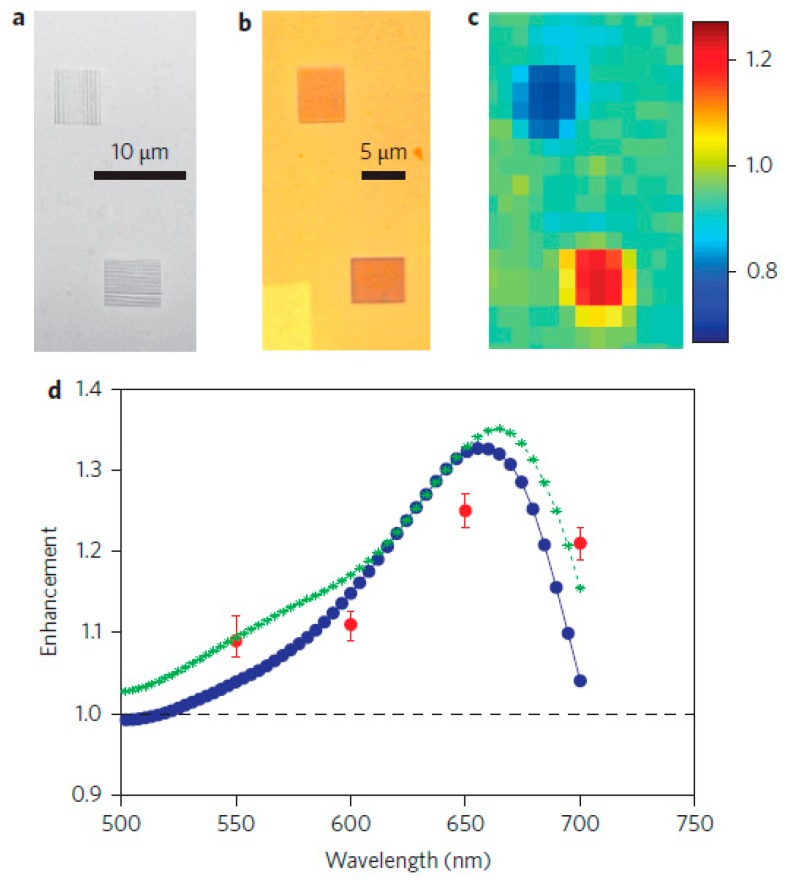
(**a**) SEM and (**b**) optical image of silver film with vertical direction (top) and horizontal direction (bottom) groove arrays. (**c**) Image showing the distribution and size of photocurrent. (**d**) Plot of photocurrent enhancement factors (blue line). The green line is obtained by replacing each original groove with 10 nm narrow grooves. The red points are real photocurrent enhancement factors, and the error bands are the maximum and minimum photocurrent values in the groove array. Reproduced with permission from [86]. Copyright Springer Nature, 2014.

Figure 6 illustrates the advantages of the MMs using photocurrent. The silver film is patterned with vertical (top) and horizontal (bottom) groove array in Figure 6a,b. Figure 6c shows the distribution and size of the photocurrent obtained. The polarized regions exhibit different properties. The photocurrent in the horizontal groove (red) is higher than the vertical (blue). This indicates that the properties of the MMs can be changed by adjusting the groove orientations. The photocurrent enhancement factor is shown in Figure 6d, and the peak is at about 650 nm (blue line). The green curve is obtained by replacing each original groove with 10 nm narrow grooves. The red data points are real photocurrent enhancement factors measured in the experiment, and the error bands are the maximum and minimum photocurrent values in the grooved area.

**Figure 7:**
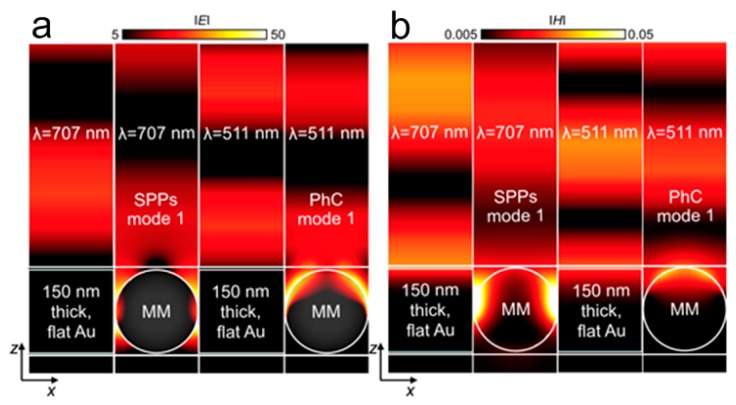
(**a**) Electric and (**b**) magnetic field distributions of flat Au layer and AuNPs array. Reproduced with permission from [87]. Copyright Optical Society of America, 2015.

Magnetic mirrors with grooves perform well, but there are some practical difficulties, such as large area fabrication and compatibility with other optoelectronic devices. Seungwoo Lee et al. [87] proposed MMs combining Au nanoparticles (AuNPs) and dielectric materials, which can achieve the effect of a magnetic mirror. High surface impedance of spherical AuNPs monolayer within a dielectric matrix (poly real-4-vinylphenol, cPVP) can effectively reduce the surface current and conductivity, enhancing the surface electric field [88,89]. Both SPPs and photonic (PhC) modes of this structure can enhance the electric field. The reflection and impedance of MMs and flat Au are calculated. The plasmonic and photonic bandgaps are at the wavelength of ~707 nm and ~511 nm, respectively. The reflection spectrum of MMs shows the opposite trend to the impedance curve with the reflection peak at ~700 nm. However, 150 nm thick flat Au embedded within 160 nm thick cPVP has very low impedance with a reflectivity of 1. The electric and magnetic field distributions of the two structures are compared in Figure 7. In Figure 7a, the light reflected from the flat Au forms strong standing waves, and the surface electric field is very small, which is caused by the superposition of incident light and reflected light (reflected phase φ = π). The AuNPs array generates a large electric field on its surface and displaces the standing wave. This is due to the smaller reflected phase of the two modes, φ = π/6.3 at the wavelength of 707 nm and φ = π/7.1 at the wavelength of 511 nm. The standing wave of AuNPs array is small, which further proves the optical storage and phase change. Figure 7b corresponds to the magnetic field distribution of two structures under two different modes. The magnetic field distribution near the mirror also accords with the above analysis. 

**Figure 8:**
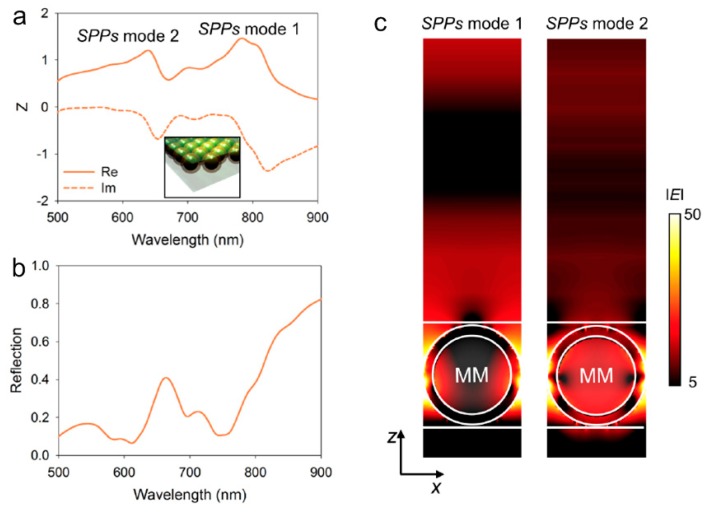
Properties of silica-Au core-shell mirrors. (**a**) Calculated impedance and (**b**) reflection of silica-Au core-shell NP array. (**c**) The electric field distribution of MMs at SPPs mode 1 and mode 2. Reproduced with permission from [87]. Copyright Optical Society of America, 2015.

In addition, the core-shell structure is designed using a silica core (120 nm in diameter) and Au shell (15 nm in thickness) NPs array embedded within cPVP matrix (the inset of Figure 8a). Since the refractive index of silicon and cPVP matrix is similar, the PhC mode disappears. There are two modes of SPPs, and the maximum impedance’s real part is 1.49 for mode 1 and 1.25 for mode 2. It can be seen from Figure 8b that the resonance wavelength moves from 707 nm to 790 nm. Meanwhile, this complex structure makes the photocurrent path zigzag, which increases impedance and reduces reflection. Compared Figure 8c with Figure 7a, the silica-Au NPs array exhibits weaker standing wave at SPPs modes. The field is mainly distributed in the vicinity of core-shell NPs rather than the surface. This field distribution is not ideal for photoelectric devices. The AuNPs with higher surface impedance exhibit stronger effect of collecting electric field than the flat Au layer. In the modified structure, the reflected phase decreases, but the electric field is not on the surface of the structure. Therefore, it is important to manipulate electric field distribution, reflection phase, and surface impedance of MMs.

## 4. Metamaterial Biosensors

Generally, the performance indices of biosensors include sensitivity, detection limit, stability and response time. In order to improve the sensitivity of biosensors, it is necessary to enhance the interaction between electromagnetic waves and samples. The special metamaterial structure and thin substrate with low dielectric constant and low loss can improve the detection sensitivity, which is beneficial to detect slight changes of materials and reduce the number of required samples.

Single-stranded oligonucleotides can fold into four-stranded G-quadruplexes (G4) in univalent ions, such as K^+^ and Na^+^, through the Hoogsteen hydrogen bond G-tetrad squares [90,91,92,93,94]. The structural changes and chemical properties in this process can be identified and detected by the transmission and Raman scattering spectra. It is suggested that G4 can be used as a capture scaffold for specific molecules [95,96]. Moreover, there is a large amount of nucleoprotein in eukaryotic cells [97], which is often over-expressed in cancer cells through RNA-specific binding regions and arginine-glycine-glycine (RGG_9_ peptide) domain [98]. Studies have also shown that the combination of G4 can affect the expression of nuclear function and inhibit the proliferation of cancer cells, making G4 potentially useful as an anticancer drug [97,99]. This section introduces two kinds of metamaterial biosensors which combine the dielectric sensing and SERS molecular recognition, thus providing convenience for the label-free conformation and quantitative detection. The experiments select G4 moieties as a plasmonic response regulator and captor for sensing of malachite green (MG).

**Figure 9:**
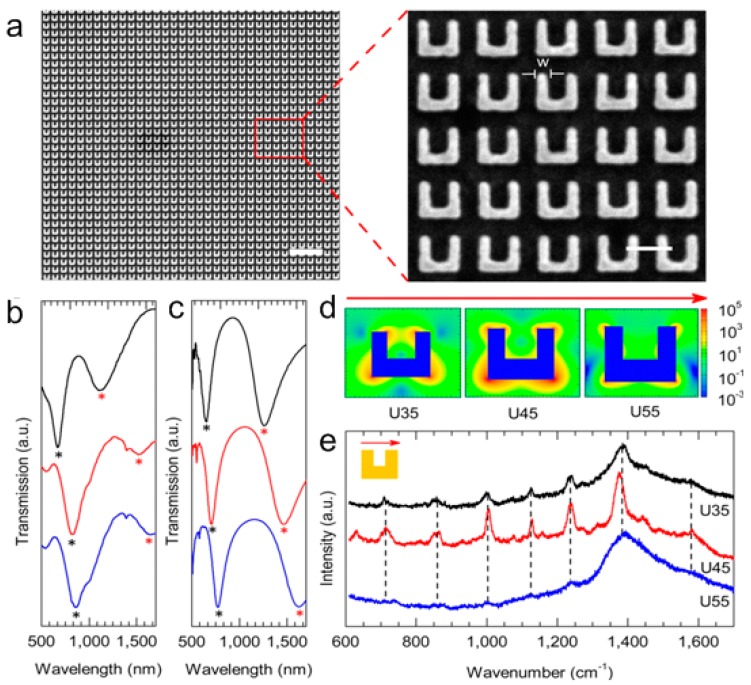
The geometry and characteristics of split-ring resonators (SRR) biosensors. (**a**) Overview SEM and magnified image of U45 SRR array. (**b**) The transmission spectra of three U-shaped SRRs measured in the experiment (U35 in black, U45 in red, and U55 in blue), compared with (**c**) Simulate transmission spectra. The electric resonance (ER) and magnetic resonance (MR) modes are marked with black and red asterisks, respectively. (**d**) Calculated local electric field (|E|^4^) of three structures. (**e**) The surface-enhanced Raman scattering (SERS) spectra of single-stranded oligonucleotides attached to three types of U-shaped SRRs. Reproduced with permission from [100]. Copyright American Chemical Society, 2013.

The first experiment shows highly tunable plasmonic metamaterials for the acquisition of optical transmission and sensitive SERS spectra, probing the conformational states and binding affinity of biomolecules in different environments. Gold U-shaped split-ring resonators (SRRs) with the width of approximately 35, 45 and 55 nm (labeled as U35, U45, U55) are defined. Figure 9a is the SEM and magnified image of the U45 SRR array. Figure 9b shows the measured transmission spectra of three SRRs (U35 in black, U45 in red and U55 in blue). There are two resonance valleys in the visible and near-infrared band, representing the electric resonance (ER) and magnetic resonance (MR), respectively. The ER is related to collective oscillation of free electrons in plasmonic structures, and the MR is resulted from loop current generated by the electrical polarization along the gap-bearing of structures [49,50,101,102,103]. 

As the width of SRRs increases, the resonant valley redshifts. Figure 9c is the simulated transmission spectra of three U-shaped SRRs, which are generally consistent with experimental results. The slight difference in resonance wavelength is caused by different parameters of the experimentally etched structures and simulated ideal cases. The ER and MR modes are marked by black and red asterisks, respectively. The sensitivity to the refractive index increases from 137 nm to 339 nm per refractive index unit (RIU) with increasing SRR width, which is similar to the traditional SRR biosensors (~100 nm to 300 nm per RIU) [35,45,104,105,106]. Each SRR nanostructure has a large local electromagnetic field, which occurs when the incident wavelength is close to the resonant wavelength [35,107,108]. In this experiment, the wavelength of 785 nm is selected as the excitation source, and its polarization direction is parallel to the gap. The local electric field (|E|^4^) can be seen from Figure 9d that the electric field mainly distributes at the corners of the structures, and the local electric field of U45 is the largest. Figure 9e is the SERS spectra of the single-stranded oligonucleotides attached to three SRRs. Compared with other curves, the peak of U45 curve (red line) is more obvious and sharper, and the electric field is larger and easier to observe under the same conditions. 

**Figure 10:**
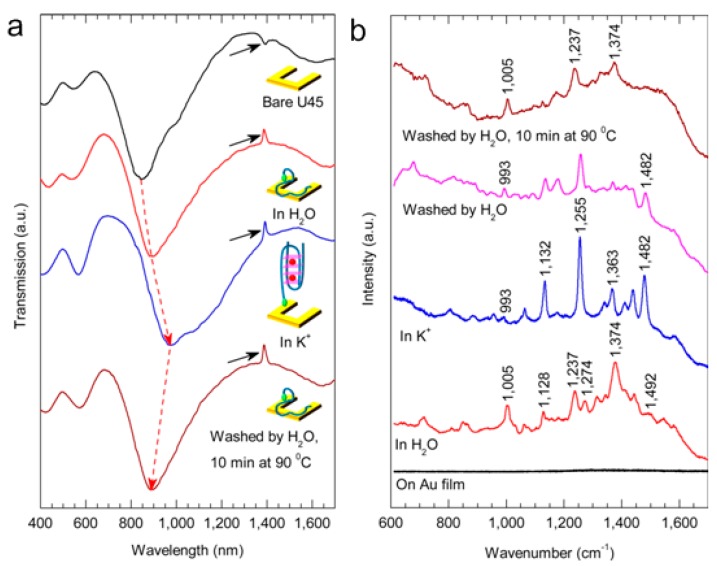
Conformation analysis of the single-stranded oligonucleotides attached to the SRRs in different states. (**a**) From top to bottom, the transmission spectra of bare U45 SRRs, fixed in thiolated single-stranded DNA in water, then folded into G4 in K^+^ buffer, and washed by water for 10 min at 90 ℃. Resonance wavelengths are 844, 895, 970 and 889 nm, respectively. The resonant peaks are marked by black arrows. (**b**) Raman spectra of the above states. The black line is Raman signal of single-stranded oligonucleotides on Au film. Reproduced with permission from [100]. Copyright American Chemical Society, 2013.

Figure 10a plots the transmission spectra of U45 in different states. From top to bottom, the ER and MR valley of the bare U45 are 844 nm and 1620 nm, respectively. When the U45 is attached to oligonucleotides and placed in water, its ER valley is shifted to 895 nm (MR valley to 1678 nm). After single-stranded oligonucleotides are put into K^+^ buffer and folded to G4, the resonance wavelengths of the two modes are 970 nm and 1700 nm (beyond the spectral range of the microspectrophotometer). In the ER mode, resonance wavelength shifts ~75 nm, due to the change of refractive index caused by the DNA structural change and K^+^ buffer. The black arrows point out the resonant peaks. 

Figure 10b compares the SERS spectra of DNA in different states. From bottom to top, there is no Raman signal detected in the single-stranded oligonucleotides on Au film (black line). In water, the spectrum has a sharp peak at 1005 cm^−1^, which is due to C-C stretch on a deoxyribose backbone [109]. The peaks of 1128 cm^−1^ and 1237 cm^−1^ are generated by unpaired dT (N3) [40,110], and the C-H deformation in thymine produces 1274 cm^−1^ peak [109]. There is a prominent peak at 1374 cm^−1^ attributed to the coincidence of C2′-endo/syn dG and C2′-endo/anti dT markers [110]. Treated with K^+^, Raman signal shows different states. There is a strong peak at ~1482 cm^−1^ caused by the marker of C8 = N7-H2 Hoogsteen hydrogen bonding of the folded G4 structure [40,109,110,111,112]. The peak of C2′-endo/syn dG shifts from 1374 cm^−1^ to 1363 cm^−1^. When G4 is washed away by water, the signal intensity decreases at 1482 cm^−1^. The absence of K^+^ leads to the loss of hydrogen bonding between N7 and H2 of C8 = N7-H2, making the G-tetrad planes loose. The structure is then processed for 10 min at 90 °C in water, and the Hoogsteen band is completely destroyed. The increasing intensity of spectrum at 993–1005 cm^−1^ means that the conformation of DNA has changed from folded G4 to single-stranded. Furthermore, the resonant valley of ER blueshifts to 889 nm, which can prove the function of U-shaped SRR metasensors.

**Figure 11:**
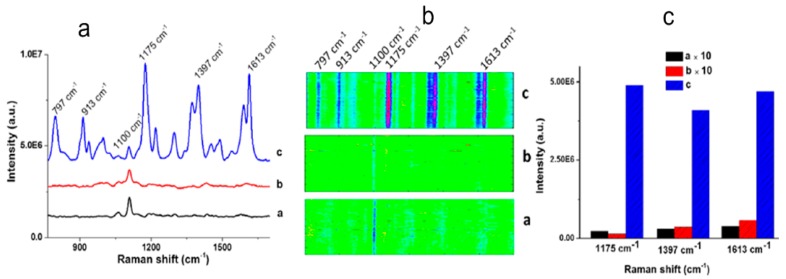
SERS spectra of different modified nanoporous gold (NPG) disks. (**a**) Spectra of MCH functionalized NPG disks immersed in malachite green (MG) solution (black curve), G4-functionalized NPG disks immersed in buffer solution (red curve) and in MG solution (blue curve). (**b**) Image of different states NPG disks. (**c**) Corresponding SERS intensity at 1175 cm^−1^, 1397 cm^−1^ and 1613 cm^−1^. Reproduced with permission from [113]. Copyright American Chemical Society, 2016.

The second experiment is a SERS sensor which can detect small molecules using G4 moieties functionalized on nanoporous gold (NPG) disks as a capturing scaffold. The feasibility of SERS sensing is verified in Figure 11. In Figure 11a, the Raman spectrum (black curve) is obtained by using 6-mercaptohexanol (MCH) to block NPG disks from the nonspecific binding of small molecules [114,115] and putting in MG solution. There is only one significant peak at ~1100 cm^−1^. 

**Figure 12:**
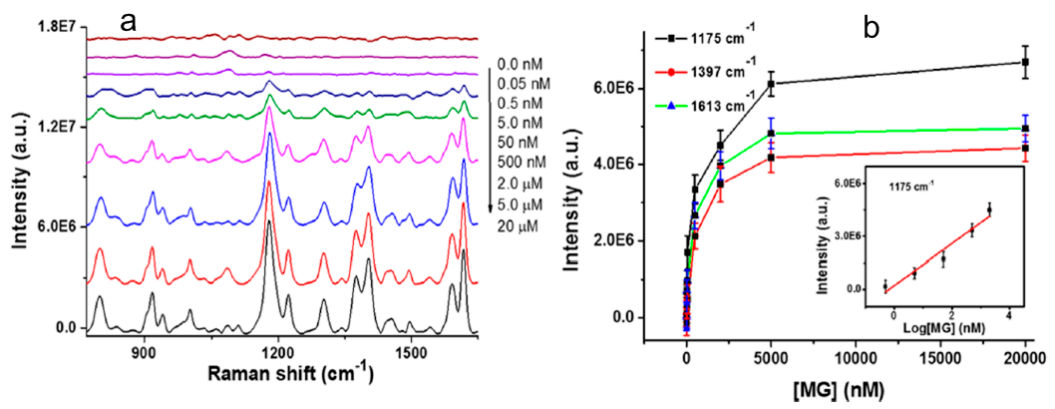
SERS spectra with different MG concentrations. (**a**) Spectra of the MG concentrations from 0.05 nM to 20 μM. (**b**) SERS intensity at 1175 cm^−1^, 1397 cm^−1^ and 1613 cm^−1^ as a function of MG concentration. Inset is a linear relationship fit between the intensity and the logarithm of MG concentration at 1175 cm^−1^ at the range of 0.5–2000 nM. Reproduced with permission from [113]. Copyright American Chemical Society, 2016.

The similar red curve is measured by immersing the G4-functionalized NPG disks in buffer solution instead of MG. The SERS spectrum of the structure in MG solutionas is plotted with blue curve. There are several obvious peaks at 1175 cm^−1^, 1397 cm^−1^, and 1613 cm^−1^, which are in-plane modes of C-H bending, N-phenyl stretching, and ring C-C stretching in MG molecules, respectively. Peaks at 797 and 913 cm^−1^ are assigned to ring C-H out-of-plane bending [116]. Using a line-scan Raman microscope to detect the corresponding images of 133 SERS spectra (Figure 11b), one can see that only a significant single peak is observed in the absence of MG or G4. The signal intensity of 1175 cm^−1^, 1397 cm^−1^, and 1613 cm^−1^ are compared in Figure 11c. The intensity of G4-NPG disks with MG is greater than that without MG or G4 moieties. Raman scattering spectra are also measured with different MG concentrations. In Figure 12a, the maximum intensity is obtained when the MG concentration increases to 5.0 μM. Figure 12b shows that SERS intensity at 1175 cm^−1^, 1397 cm^−1^ and 1613 cm^−1^ as a function of MG concentration. When the concentration is less than 5.0 μM, the intensity of each position heightens with the increasing concentration. However, when the MG concentration is more than 5.0 μM, the intensity increases slowly because the binding speed with G4 slows down. There is a linear relationship between the logarithm of MG concentration and intensity in the range of 0.5–2000 nM. Taking the intensity at 1175 cm^−1^ as an example, the diagram is shown in the inset. Local electric field is enhanced on NPG disks. G4 is highly bound to MG molecules, and the NPG disks can hold more G4 and thus capture more MG [117], making the structures more sensitive.

## 5. Metamaterial Detectors

**Figure 13:**
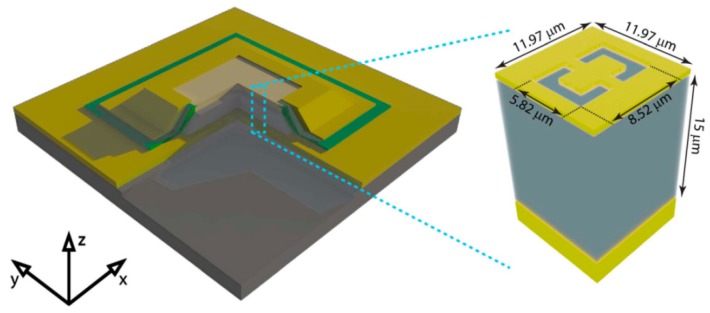
The structure of metamaterial detector. The yellow layer on the top is metamaterial region (the green part is SiN_x_ insulation). The coordinate system in the left corner represents the axis direction. Reproduced with permission from [53]. Copyright Springer Nature, 2014.

Quantum cascade devices currently are important to develop active optical devices, mainly including quantum cascade lasers (QCLs) and quantum cascade detectors (QCDs). Research on QCLs has reached a relatively mature stage, and various material systems and structural designs have been reported [118,119]. The progress of QCDs is relatively lagging behind. QCDs are composed of periodic units, each of which consists of several coupled quantum wells. The working process of QCDs is the transition of electrons from the trapped bound state to another bound state. Figure 13 shows a typical metamaterial QCD. The complementary double split-ring resonators (CSRRs) are patterned into the metallic top contact. One needs to make a particular incident frequency to resonate with the detection. On the other hand, the incident light propagating along the z direction is transferred into the z component of electric field Ez. Because only Ez can interact with the inter-sub-band transitions (ISTs) [120,121,122]. 

Moreover, the resonant frequency of metamaterials and ISTs can be modulated and the detector can cover the middle infrared and THz bands. The active region of QCDs is composed of GaAs/Al._15_Ga._85_As heterogeneous structures. The entire substrate is covered by a thin metallic sheet. The response rate of the introduced detector is slightly lower than that of the most advanced quantum well-infrared detectors [123,124,125,126,127,128,129].

**Figure 14:**
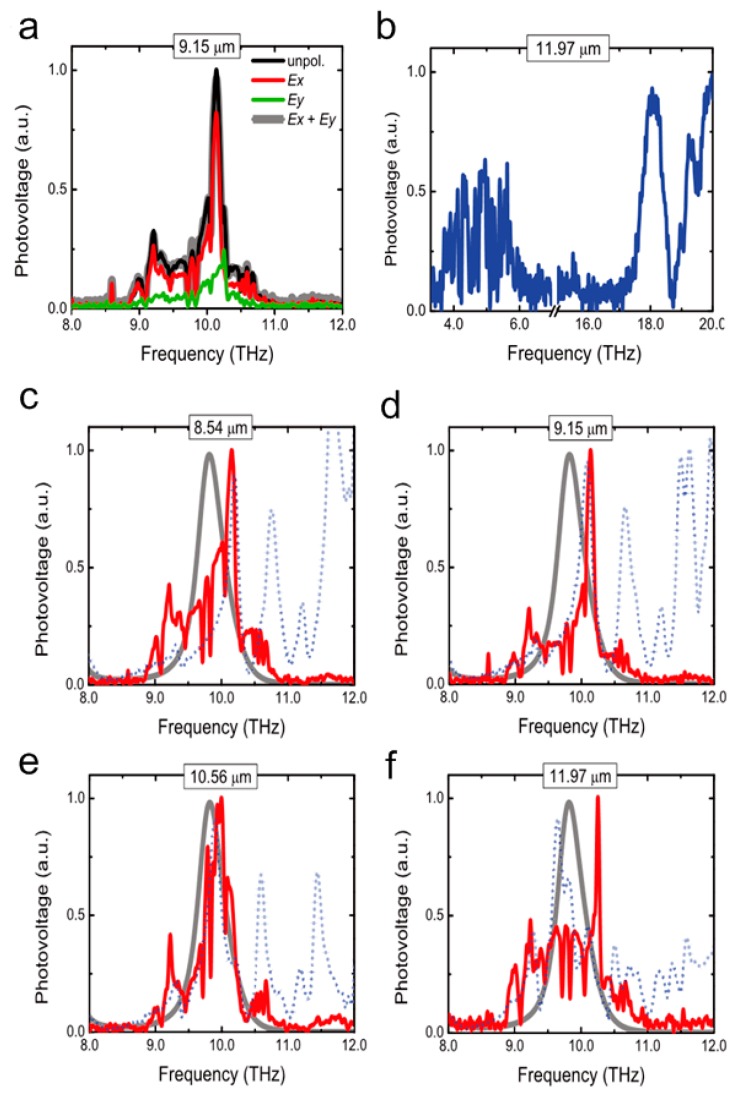
Comparison of the response spectra between experimental and simulation results. (**a**) The spectra with a period of 9.15 μm in different polarization directions. (**b**) Spectrum with period of 11.97 μm of the unpolarized response at low and high frequencies. (**c**–**f**) The optical responses of the experiment (red) and simulation results (blue dotted) of period 8.54, 9.15, 10.56, and 11.97 μm at the Ex polarization direction. The gray line represents the inter-sub-band transition. Reproduced with permission from [53]. Copyright Springer Nature, 2014.

**Figure 15:**
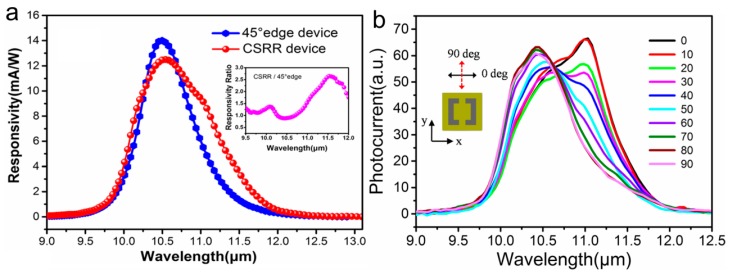
(**a**) Photocurrent responsivity of the SRRs coupled device and 45° edge facet coupling device. (**b**) The photocurrent spectra as a function of wavelength at different polarization angles. Reproduced with permission from [54]. Copyright SpringerOpen, 2016.

The reflection and transmission characteristics depend on the size and shape of the metallic resonators. The CSRRs with periods of 8.54, 9.15, 10.56, and 11.97 μm are fabricated. Firstly, the optical characteristics with a period of 9.15 μm in different polarization directions are introduced (Figure 14a). For Ex polarization (red), there is a visible peak at ~10.14 THz, which is different from the Ey polarization (green) whose peak is at 10.25 THz and weaker. Figure 14b presents the spectrum with the period of 11.97 μm of the unpolarized response at low and high frequencies. It is corresponding to the transition between state 1 and state 2 (there are four main bound-to-bound transitions in active region) with the transition energy of 16.8 meV (= 4.1 THz). The peak at 18 THz is related to the transition between higher lying states in the structure (higher carrier concentration at the top and the bottom of quantum-well stack). Moreover, there is a peak at 20 THz presenting the last bound-to-bound transition with the transition energy of 88.9 meV. Figure 14c–f compare the optical responses of experimental detectors with the simulation results at the Ex polarization direction. The detector with period of 8.54 μm has two peaks at 10.16 THz and 9.2 THz, which are basically the same with the simulation results. Changing the period to 9.15 and 10.56 μm, it can be found that the peaks both redshift to 10.14 and 9.95 THz. Same changes are observed in simulations and the shapes of lines are similar to the experimental results. The curve with period of 10.56 μm tends to be symmetric, while the response range of period 11.97 μm is very wide with a sharp peak at 10.25 THz. The line′s behavior is consistent with the metamaterial resonance at 10.1 THz, but the amplitude of experiment is higher than the simulation. This is mainly due to the differences in the structure parameters between etched structures and the ideal model, e.g., the inhomogeneity of refractive index. Wang et al. [54] proposed similar QCDs which exhibit significant enhancement of photocurrent response and polarization dependent property with normal incidence. The InGaAs/InAlAs active region and two Si-doped InGaAs contact layers are sandwiched between an upper Au metamaterial layer and a bottom Au reflection layer. Due to the asymmetry, the SRRs have different resonant properties for the incoming light with different polarized directions. The measured resonance wavelengths are 10.7 and 11.5 μm for Ex polarization incident light and 10.3 μm for Ey. In Figure 15a, the photocurrent responsivities of the metamaterial detector and a 45° edge facet coupling device are measured. The SRR coupled device is clearly modified compared to the 45° edge facet coupling device. The largest response enhancement factor is ~165% at 11.5 μm for Ex polarization. The inset is the responsivity ratio of the SRR device to 45° edge facet coupling device. After calculating the total Ez amplitude for both Ex and Ey polarization incident light at the resonance wavelength of 10.7 and 10.3 μm, the former has a larger electric field, which further proves the Ex polarization incident light can bring stronger response enhancement. Then the polarization-dependent property is gained by measuring the photocurrent in different polarization directions in Figure 15b. Zero degree polarization corresponds to polarization in x direction, as shown in the inset. When the polarization angle is 0° and 90°, the photocurrent is relatively strong. The spectra show a tendency to blue-shift as the polarization angle increases. 

## 6. Conclusion and Outlook

We have reviewed several different metamaterial-based devices, including near-field energy transfer components, metamaterial mirrors, metamaterial nanobiosensors, and QCDs. The unique near-field heat transfer provides a theoretical basis for extensive applications in fields such as thermal photovoltaic and radiation refrigeration. Metamaterial mirrors provide a new method for enhancing the light absorption. Based on this concept, the devices that can enhance the response to randomly polarized light will be further developed. Electromagnetic metamaterials in biosensor research have made certain progress, which can be mainly reflected from the structure of special materials design, optimization of the substrate, and specific combination of functional materials. QCDs enable photo-excited electrons to spontaneously transport in one direction, providing convenience for the transport and collection of photoelectric signals. Moreover, it only generates light current in the presence of light, so the power consumption of the device is very low. Based on these advantages, QCDs have become a promising infrared detector in the fields of satellite remote sensing, laser communication, and infrared imaging. More specific and special potential applications and functions of metamaterials remain to be further excavated. New materials will become an important entry point and direction of nanophotonics research. At present, the development of practical applications based on these experimental results is also accelerating. More designs and applications of metamaterials can provide a driving force for exploring new functions of materials, leading to new directions for industry or improving comprehensive properties of materials and breaking through the bottleneck of scarce resources.
